# The COVID-19 pandemic through eyes of a NYC fertility center: a unique learning experience with often unexpected results

**DOI:** 10.1186/s12958-020-00663-3

**Published:** 2020-11-04

**Authors:** Norbert Gleicher

**Affiliations:** 1 The Center for Human Reproduction, 21 East 69th Street, New York, N.Y 10021 USA; 2 The Foundation for Reproductive Medicine, New York, N.Y USA; 3 Stem Cell Biology and Molecular Embryology Laboratory, The Rockefeller University, New York, N.Y USA; 4 Department of Obstetrics and Gynecology, Medical University of Vienna, Vienna, Austria

**Keywords:** COVID-19 and infertility, Herd immunity, COVID virus strains, Infectivity, Innate immunity

## Abstract

Affecting basic tenets of human existence such as health, economic as well as personal security and, of course, reproduction, the COVID-19 pandemic transcended medical specialties and professional disciplines. Yet, six months into the pandemic, there still exists no consensus on how to combat the virus in absence of a vaccine. Facing unprecedented circumstances, and in absence of real evidence on how to proceed, our organization early in the pandemic decided to act independently from often seemingly irrational guidance and, instead, to carefully follow a quickly evolving COVID-19 literature. Here described is the, likely, unique journey of a fertility center that maintained services during peaks of COVID-19 and political unrest that followed. Closely following publicly available data, we recognized relatively early that New York City and other East Coast regions, which during the initial COVID-19 wave between March and May represented the hardest-hit areas in the country, during the second wave, beginning in June and still in progress, remained almost completely unaffected. In contrast, south western regions, almost completely unaffected by the initial wave, were severely affected in the second wave. These two distinctively different infectious phenotypes suggested two likely explanations: The country was witnessing infections with two different SARS-CoV-2 viruses and NYC (along with the East Coast) acquired during the first wave much better immunity to the virus than south western regions. Both hypotheses since have been confirmed: East and West Coasts, indeed, were initially infected by two distinctively different lineages of the virus, with the East Coast lineage being 10-times more infectious. In addition, immunologists discovered an up to this point unknown long-term anti-viral innate (cellular) immune response which offers additional and much broader anti-viral immunity than the classical adaptive immunity via immobilizing antibodies that has been known for decades. Consequently, we predict that in the U.S., even in absence of an available vaccine, COVID-19, by September–October, will be at similarly low levels as are currently seen in NYC and other East Coast regions (generally < 1% test-positivity). We, furthermore, predict that, if current mitigation measures are maintained and no newly aggressive mutation of the virus enters the country, a significant fall-wave of COVID-19, in combination with the usual fall wave of influenza, appears unlikely. To continue serving patients uninterrupted throughout the pandemic, turned for all of our center’s staff into a highly rewarding experience, garnered respect and appreciation from patients, and turned into an absolutely unique learning experience.

## Introduction

As of July 9, the U.S. experienced a total of 3,094,776 diagnosed COVID-19 cases, with 62,893 added in the preceding 24 h alone, and mostly reflective of an ongoing second, late wave in south western states that had only started in early July. The national mortality rate stood as of that date at 132,166, with 987 cases added on the preceding day, representing a 4.27% relative mortality rate in diagnosed COVID-19 patients [the term “relative” applies since mortality rates reflect only known cases of COVID-19. Those, however, may represent only ca. 10% of total cases, with many being asymptomatic or only mildly symptomatic [1]. Based on most recent media reports, the U.S. has so-far seen in excess of 5.000.000 known cases by August 15, with deaths exceeding 160,000 (The New York Times recently claimed that the factual death rate may already exceed 200,000, August 14). Relative mortality in August of approximately 3,2% is, therefore, significantly lower than July data. Though not widely reported, this observation suggests declining viral virulence in the still ongoing second (here also called later) wave. Rates for individual states are accessible at https://usafacts.org/visualizations/coronavirus-covid-19-spread-map/, a database also utilized by the Centers for Disease Control and Prevention (CDC) as a basis for national COVID-19 reporting.

### A brief timeline

The severe acute respiratory syndrome coronavirus 2 (SARS-CoV-2) which causes coronavirus disease 2019 (COVID-19) is the seventh coronavirus known to infect humans [2] and the third causing disease that co-opts the peptidase angiotensin-converting enzyme 2 (ACE2) in order to achieve entry into cells [3]. Like all coronaviruses, this one also gains access through its 18-kDa spike (S) protein by binding to ACE2 and through fusion of viral and cellular membranes. A very recent phylogenetic analysis of SARS-CoV-2 in Northern California discovered at least seven different cell lineages of the virus, with lack of dominance of any one among them. Single base substitutions in the viral genome were associated with individual outbreaks [4]. Considering this large number of cell lineages in only a small part of the U.S., the virus can be expected, to demonstrates even more genomic variability on a national scale.

Historically, the first U.S. COVID-19 case was diagnosed in Washington State on January 19, 2020 [5] (Table 1). The virus likely circulated in the local population already weeks earlier. On the East Coast in New York City, the first case was diagnosed on March 1 [6]; based on traced-back blood samples, the virus, however, circulated in the city already by late January [7]. On both coasts the virus rapidly entered the community. West and East Coasts, however, appeared to develop very distinct epidemic phenotypes, widely interpreted to reflect better and poorer management skills of the pandemic by local governments [8].

**Table 1:** A brief timeline of COVID-19 in New York state^a^

Date in 2020	Event	Cases (n) in NY-state	Deaths (n) in NY-state
Sometimes in December	First COVID-19 cases in Wuhan, China		
1/17	1st U.S. cases on West Coast		
3/1	1st case in NYC	1	0
3/7	State of emergency in New York state	76	0
3/10	1st regional quarantine in New Rochelle	173	0
3/12	Prohibition of gatherings >500 people	325	0
	Broadway closes		
3/14	First deaths	613	2
3/16	Governor issues work from home order for non-essential workers; schools and movie theaters closed	950	7
3/17	1st ASRM GUIDANCE		
3/18	Non-essential retail stores closed	2382	16
3/20	All non-essential businesses closed NY state “on pause”	7102	46
4/1		83,712	1,957
4/6		139,689	4,774
4/12		188,694	9,401
6/1	Riots in NYC		
By July			
NY state		~390,000	~31,000
NYC		~ 214,500	~21,000

^a^Approximately two-third of all cases occurred in NYC

**Fig. 1:**
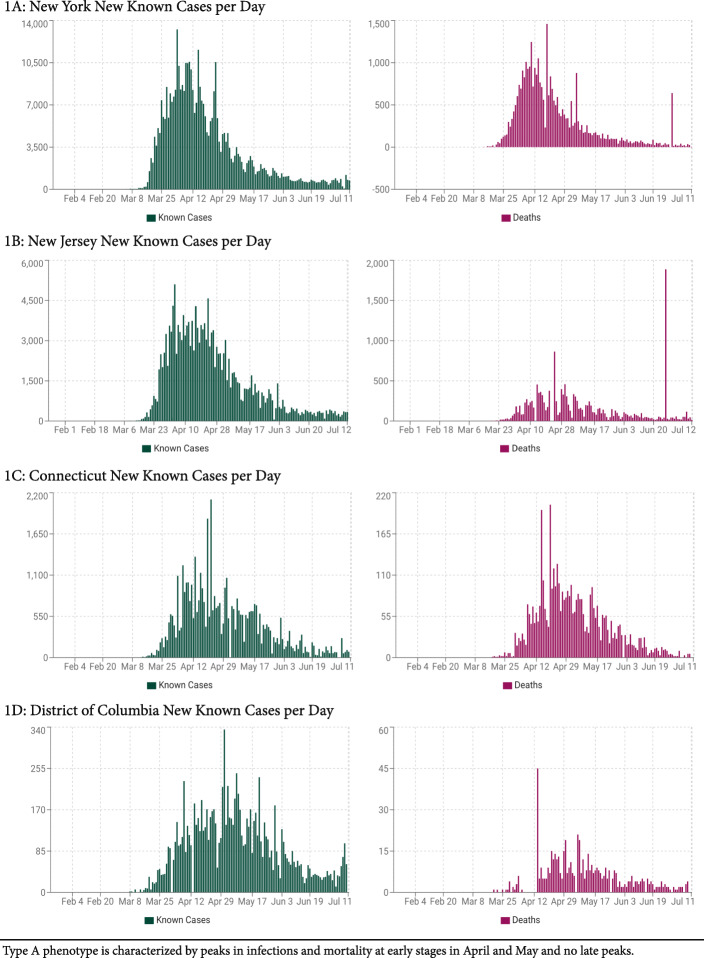
Typical infection and mortality rates in selected Type A states

**Fig. 2:**
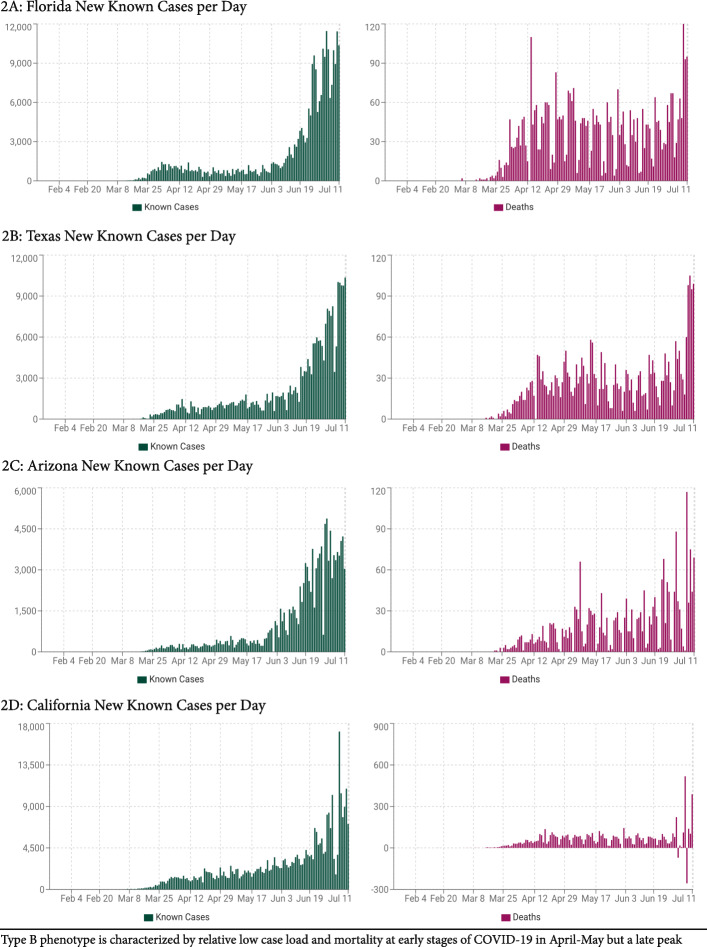
Typical infection and mortality rates in selected Type B states

**Fig. 3:**
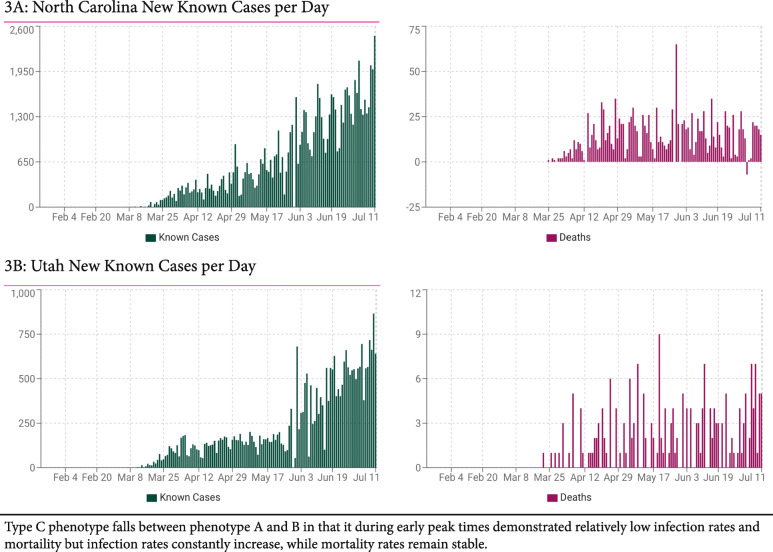
Typical infection and mortality rates in selected Type C states

Following national infection and mortality rates in different U.S. states, we were not convinced of this explanation: Characterized by much lower infection and mortality rates, the pandemic in south western states during early stages (peaks in April–May) appeared to have a very different epidemic signature in comparison to East Cost states. Tabulating the differences, we defined three distinct epidemic phenotypes, here for the first time describe in print phenotypes, A (East Coast), B (south west) and C (mixed pattern) (Figs. 1, 2 and 3). With extended epidemiological observation, those differences between states became even more obvious because, starting in June and reaching peaks in July, a second wave of infections hit the U.S., though this time in opposite directions: East Coast now demonstrated only minimal, indeed almost no new disease, while south western states, suddenly, reported explosive growth in case numbers, and modest increases in hospitalizations and mortality. Figures 1, 2 and 3 and Table 2 demonstrate sample states for all three epidemic phenotypes. Table 2 also demonstrates, the mortality between A and B (as well as C) differed significantly (*P* < 0.0001), suggesting different infectivity and virulence of COVID-19 between these epidemic phenotypes. How that was possible, at that point was, however, still unclear.

**Table 2:** COVID-19 characteristics of selected U.S. states as of July 9, 2020

State	Number of cases	Number of deaths	%^*^	Designation pattern	Peak timing^a^
New York	399,575	31,980	8.0	A	early
California	300,324	6,849	2.3	B	late
Florida	232,718	4,009	1.7	B	late
Texas	230,346	2,918	1.3	B	late
New Jersey	174,628	15,479	8.9	A	early
Illinois	150,450	7,119	4.7	A	early
Arizona	112,671	2,038	1.8	B	late
North Carolina	79,349	1,460	1.8	C	none
Connecticut	47,209	4,348	9.2	A	early
Utah	27,519	202	0.7	C	none
District of Columbia	10,743	568	5.3	A	early

^*^The difference in relative mortality rate between Type A and Type B states, as well as between Type A and Type A+B states was highly significant (*P* < 0.0001), with Type A states experiencing significantly higher mortality

^a^“Early,” denotes disease peaks in April and May; “late,” denotes disease peaks in June and July

Answers dribbled in only slowly, with above noted study of viral cell lines in a small part of California [4] only confirming earlier clues: COVID-19 apparently reached the U.S. from two directions, through a virus strain coming with air travel directly from Asia (probably China) to the West Coast, while the East Coast primarily fell victim to travel from Europe, arriving in the U.S. mostly via New York City’s airports [8]. Having mutated in Europe from the so-called D614 to the G614 strain, the virus was now 10-times more infectious than when it originally left China [9] and, therefore, 10-times more infectious that the virus that, two weeks earlier, had hit the West Coast. How this single mutation in the virus affected its virulence has to this date not been clearly determined. Comparing mortality rates between East and West Coasts (Table 2, demonstrates lower infection and mortality, *P* < 0.0001 in B and C states in comparison to A states during the first phase of the pandemic in April and May), however, strongly suggests that with infectivity, virulence increased in parallel. Early differences in COVID-19 behavior between states, therefore, likely had little to do with political talent and everything with the biological behavior of two distinctly differently behaving virus lineages.

Though Figs. 1, 2 and 3 are extracted from publicly available data at https://usafacts.org/visualizations/ coronavirus-covid-19-spread-map/), these observations, to the best of our knowledge, have not been previously interpreted in this way. Their relevance may also extend beyond the U.S. to countries that mimic A and B state patterns. Examples are Japan and Israel, during early stages of the pandemic widely lauded for their successful management but, in the second wave that initiated in July, like B states, severely affected by disease.

With A states after May continuing to demonstrate declining disease rates, hospitalizations and mortality, starting in June, B and C states demonstrated the opposite. States like Florida, Texas, Arizona and California, suddenly, experienced what New York, New Jersey and Connecticut had already experienced during the initial wave in April and May, though with one difference: while infectivity was very similar, virulence fortunately has been less severe, causing, therefore, much lower mortality than the G614 lineage had caused on the East Coast. Our interpretation of the second wave in south western states has been that, in an evolutionary sense, G614 had beaten out D614 in its earlier territories. Because D614 had, however, during earlier months established a low level of immunity in the population in those states, the disease was milder. Data also suggested that patient populations admitted to hospitals in the later wave were younger, which also could have contributed to lower mortality.

New York City (NYC), at one time the most significant hotspot of COVID-19 in the world, toward the end of June and into early July also witnessed the largest political unrest of any major city in the U.S. with participants in demonstrations and other political fall out hardly ever complying with distancing and face masking rules. Though there was considerable concern that these activities would lead to a spike in COVID-19 infections, most remarkably, nothing happened. Infection rates in the city, hospitalizations and deaths, indeed, continued to decline, like none of above noted breaches had ever happened. On July 12, New York City for the first time recorded not even a single COVID-19 death.

In this case, most media attributed the continuous suppression of COVID-19 in city and state to the delayed reopening of the economy ordered by city and state governments. We, however, again offer an alternative explanation: Above noted biological differences in infectivity and virulence between the D614 and G614 lineages, likely are also reflected in the immune responses these two mildly different virus lineages induced. The G614 strain, therefore, likely induced immunity more aggressively than the D614 strain. New York City, consequently, already in March through May, likely, gained significantly better immunity to the virus than B and C states. There was simply no other explanation for why B and C states by June started to get severely sick, while East Coast states generally continued in their respective declines. Was it possible that New York City and other A states/regions had reached already some kind of herd immunity that protected the population from further COVID-19 infections? Please also note the added note of proof at end of the manuscript.

## The concept of herd immunity

Mayo Clinic investigators recently described herd immunity as occurring, *“when a large portion of a community (the herd) becomes immune to a disease, making the spread of disease from person to person unlikely”* (https://www.mayoclinic.org/diseases-conditions/coronavirus/in-depth/herd-immunity-and-coronavirus/art-20486808). In developing public policy against COVID-19, herd immunity has remained a highly controversial subject. Sweden, one of only few countries pursuing herd immunity, experienced higher mortality than neighboring countries with alternative policies that more approximated the U.S. model of economic shut down and school closures. Though the Swedish model of keeping significant portions of the economy and of schools open with time appeared to competitively improve, a final verdict about strategies against COVID-19 is still pending [10]. At least initially, however, most “experts” declared the Swedish response to COVID-19 to be inferior [11].

The understanding of what represents adequate herd immunity is, however, quickly changing. Until recently, the undisputed assumption was that in order to reach herd immunity at least between 60 and 70% of a population must have neutralizing antibodies against a virus. Further assumptions were that only such antibody responses by the adaptive immune system were able to establish immune responses with long-term memory and that such antibodies could be obtained by individuals only through being infected or via vaccination. Under these by now clearly outdated assumptions, it should not surprise that Spanish investigators, even very recently, still suggested that reaching herd immunity against COVID-19 was impossible. They reached this conclusion since only 5% of Spaniards demonstrated neutralizing antibodies to the SARS-CoV-2 virus, even though Spain was one of the most affected countries by the pandemic in Europe [12]. Similarly, in Sweden, where infections of low risk populations were almost encouraged in attempts to reach herd immunity, only 7.3% of Stockholm’s inhabitants developed antibodies [13]. And in New York City, even at the peak of the pandemic in April and May, only slightly above 20% of Manhattanites (where peak infection levels were recorded) demonstrated antibodies [14]. Interestingly, The New York Times recently reported that 68% of individuals at a testing site in Queens tested positive [15].

As only very recently published research, however, convincingly demonstrated, long-term immunity to viruses is not only the consequence of an antibody response to the virus by the adaptive immune system, as has been for the longest assumed. The more ancient part of our immune system, the so-called innate immune system, also significantly contributes to antiviral immune responses and does so primarily via cellular responses and the release of cytokines. This conclusion is supported by several recent discoveries: For example, viruses elicit a non-specific general T-cell response which results in non-specific cross-reactive immunity toward a variety of viruses. Gallo of HIV fame, therefore, recently proposed immunization with Salk’s oral anti-polio vaccine as an interim strategy to bridge the time until a specific anti-COVID-19 vaccine comes to market [16]. Bacille Calmette-Guerin (BCG) and tuberculosis endemicity have also been associated with decreased prevalence of COVID-infections [17].

A very recent study mapped 142 T cell epitopes across the SARS-CoV-2 genome and interrogated the SARS-CoV-2-specific CD4+ T cell repertoire [18]. What the investigators found was that several different preexisting CD4+ T cells (antibody-producing helper T cells) were also cross-reactive with comparable affinity to common cold coronaviruses HCoV-OC43, HCoV-229E, HCoV-NL63 and HCoV-HKUI. The authors concluded that, *“variegated T cell memory to coronaviruses that causes the common cold may underlie at least some of the clinical heterogeneity observed in COVID-19 disease.”* In other words, immunity we gathered in the past during common colds and flu episodes, may explain why, with identical exposure, some people do, and others do not, get infected with the SARS-CoV-2 virus.

Preceding this study only by a few weeks, another group of investigators had, indeed, already reported similar results: In that study, COVID-19 patients in recovery from active diseases were found to have SARS-CoV-2 specific CD8+ MHC class I-restricted T cells, including cytotoxic and suppressor T cells as well as CD4+ T cells. Moreover, individuals with absolutely no history of exposure to the virus also frequently demonstrated such cellular immunity against the virus [19]. In other words, they, too, demonstrated cross-reactive innate immunity against SARS-CoV-2 virus, acquired from innate immune responses to other viruses, likely including common cold coronaviruses and earlier SARS-infections that produced T-cell recognition long before COVID-19 appeared on the scene. Both groups’ findings, of course, also reemphasize the wisdom of annual flu vaccinations in absence of a specific anti-COVID-19 vaccine.

A still unreviewed third study also makes the point and is so-far only posted on bioRxiv, the Cold Spring Harbor Laboratory preprint server for biology [20] but should be in print soon, considering the importance of reported findings. Here, too, acute phase SARS-CoV-2-specific T cells with highly activated cytotoxic phenotype were confirmed present which, indeed, reflected markers of disease severity in patients affected by COVID-19. During convalescence T cells became polyfunctional and demonstrated a stem cell-like memory phenotype. Most remarkably, virus-specific T cells were, however, also found in family members of infected individuals who, themselves, were seronegative as well as in asymptomatic infected individuals or patients with only a history of mild disease. Quoting the authors, they concluded that, *“SARS-CoV-2 elicits robust memory T cell responses akin to those observed in the context of successful vaccines, suggesting that natural exposure or infection may prevent recurrent episodes of severe COVID-19 also in seronegative individuals.”*

In contrast to longstanding dogma that assigned long-term antiviral immunity only to the adaptive antibody responses against a virus, these new studies performed in trying to understand COVID-19 better, demonstrated beyond reasonable doubt that the innate immune system, via cellular immune responses, also significantly contributed to long-term antiviral immunity. Immunity to the SARS-CoV-2 virus, therefore, is not, as has been believed for the longest time, only reflected in IgM and IgG antibodies against the virus but also in cell-mediated immunity, for which, as of this point, no commercial diagnostic tests yet exist. Combining these two specific long-term immune responses and viewing them as a single unified and integrated immune response, already existing immunity to the SARS-CoV-2 virus may be significantly higher than has been appreciated in above noted antibody-only studies in Spain [12], Stockholm [13] and New York City [14].

In areas of the country (and elsewhere) where COVID-19 has been active with strongly immunogenic lineages, adequate levels of immunity therefore have, likely, already been reached. New York City is a good example, as the city since July continuously only demonstrates low disease levels. That A states remained mostly unaffected by the later wave of disease that was sweeping mostly south western B states, simply allowed for no other good explanation. Finding confirmation in above noted studies was then, indeed, reassuring since they offered a scientific explanation for a historical observation that, before, had no medical explanation. These observation, however, also offer good news and new hope since, barring significant new mutations in the virus, they further suggests that the worst of the COVID-19 pandemic is, likely, behind us and by year's end, the country in most states will have reached adequate immunity to maintain very low disease activity, as NYC has been witnessing. Once the current second wave, primarily affecting south western B and C states abates, this goal should be reached, even in absence of a clinically availability of a vaccine.

NYC’s current low rate of infection, therefore, very likely, once again, is not consequence of particularly “smart” health care policies pursued by city and state, but of nature’s usual defense, − the development of adequate immunity. Delaying the reopening of schools and the economy may, actually, adversely affect development of adequate immunity. Moreover, the widely feared additional wave of COVID-19 and flu-infections in the winter appears less likely, especially if an effective influenza vaccine is offered this year. That cellular immunity can play a significant role in preventing viral diseases has recently also been recognized in conjunction with HIV [21].

Considering potential population heterogeneity and likelihood of above noted T cell immunity, Swedish investigators recently published a new mathematical model for herd immunity to COVID-19, concluding that the virus, based on antibody positivity, may only require around 43% community spread [22]. Above cited studies [18–20], however, suggest that even this model may still exaggerate the required level of antibody immunity, assuming there exists in parallel a reasonable level of cellular-long-term immunity.

## The issues COVID-19 specifically presented to the infertility field

For our fertility center, the corona virus outbreak came for the first time into focus on March 13 (https://www.centerforhumanreprod.com/fertility/covid-19-and-ivf-chrs-always-up-to-date-guide/). By June, the amount of new information surrounding the virus had exploded and monthly updates were no longer adequate. The center, therefore, initiated so-called COVID-19 Bulletins that appeared on a per- need basis till July, by which time New York City’s caseload had started abating. Subjects discussed in the remaining sections of this manuscript were during heydays of the pandemic in NYC timely addressed on the center’s website, where they are still accessible (https://www.centerforhumanreprod.com/fertility/covid-19-and-ivf-chrs-always-up-to-date-guide/). We here offer shortened and updated versions.

### The decision to shut down most fertility services

Table 1 summarizes the timetable of how the COVID-19 crisis affected NYC and, therefore, our fertility center. The table demonstrates the explosive nature of the infection in city and state. A pivotal date was March 17 when the American Society for Reproductive Medicine (ASRM), fertility field’s principal professional organization in the U.S., issued a first guidance headlined, Guidance on Patient Management and Clinical Recommendations during the Coronavirus (COVID-19) pandemic (https://www.asrm.org/news-and-publications/news-and-research/press-releases-and-bulletins/asrm-issues-new-guidance-on-fertility-care-during-covid-19-pandemiccalls-for-suspension-of-most-treatments/). This guidance *“called for suspension of most (infertility) treatments,”* including ovulation induction, intrauterine inseminations, retrievals and frozen embryo transfers, as well as non-urgent gamete cryopreservation (Table 3).

**Table 3:** Initial ASRM Guidance regarding the COVID-19 pandemic

R E C O M E N D A T I O N S	
• Suspension of initiation of new treatment cycles, including ovulation induction, intrauterine inseminations, retrievals and frozen embryo transfers, as well as non-urgent gamete cryopreservation.	
• Strongly consider cancellation of all embryo transfers, whether fresh or frozen.	
• **Continue to care for patients who are currently ‘in-cycle’ or who require urgent stimulation and cryopreservation.**	
• Suspend elective surgeries and non-urgent diagnostic procedures.	
• Minimize in-person interactions and increase utilization of telehealth.	

Our interpretation of the bolded recommendation led us to reject the idea of shutting down the center

Most U.S. IVF centers in response immediately shut down, at times even leaving patients stranded in midst of cycles. On the same date, the European Society for Human Reproduction and Embryology (ESHRE), ASRM’s European counterpart, took an even more extreme position by publishing a guidance recommending that *“all fertility patients considering or planning treatment, even if they do not meet the criteria for COVID-19 infection, should avoid becoming pregnant at this time”* (https://www.eshre.eu/Press-Room/ESHRE-News#COVID19_April2).

Because what we considered its subtlety, we mostly agreed with the ASRM guidance but considered the ESHRE guidance excessively harsh. Most U.S. colleagues, however, apparently, had missed what we perceived as subtlety, understanding that the guidance automatically mandated closure of all IVF center activities. Our interpretation, in contrast, was based on the ASRM document potentially excluding “*urgent stimulations*” from recommended discontinuation of fertility services, yet, leaving undefined and, therefore, up to patients and physicians, what *“requiring urgent stimulation”* really meant (Table 3).

Our center has been serving a worldwide patient clientele for over a decade as a center of last resort. In practical terms this means that over 90% of presenting patients previously failed IVF cycles, often multiple times at multiple IVF centers. The center’s patients are, therefore, by far the oldest among all reporting IVF centers in the U.S. (median age 43 years over the last 3 years), reflecting a need for “urgency” in their clinical care. That argument alone, we concluded, excluded our center from considering a shutdown. There was, however, also a second important argument that supported such a conclusion: When three days after publications of ASRM and ESHRE guidances, New York state’s governor mandated closure of all non-essential businesses, we considered our center’s services “essential;” not only because of our unique patient population but, also because most other fertility centers by that point already had decided to temporarily close their doors. Patients, therefore, had hardly anywhere to turn to, if they considered their situations as “urgent.”

Our center at that point instigated strict mitigation policies including the following: Access to the center was restricted only to patients (no family members) and essential medical staff. Staff that could perform their work from home, were instructed to do so. Essential staff worked in two shifts, should one shift get contaminated, the other could take over. Staff members were, furthermore, instructed to avoid public transportation and the center assumed all addition transportation costs, including garage costs for personal vehicles. Plexiglass barriers were put in place in the reception area, even though even patient access had been restricted to monitoring and procedure visits only. All consultations were conducted virtually, which for our center did not represent a big adjustment since over half of our center’s patients traditionally have been long-distance patients from outside the larger NYC tristate area for whom virtual consultations have been routine for years. Anybody entering the facility underwent a temperature check and was asked a serious of specific questions regarding possible COVID-19 symptoms. Luckily, the center did not experience even a single COVID-19 case among staff or patients during that period of heightened concerns. One staff nurse fell ill with the virus while already for two weeks on a leave of absence.

On April 7, New York’s governor reaffirmed our opinion about the essentiality of fertility services in a guidance from the state’s Department of Health that specifically defined, “*fertility services, including infertility treatment and procedures,”* as essential services, *“with the ultimate decision on when such services must occur to be between patient and clinical provider”* [23]. NYC’s Center for Human Reproduction (CHR), therefore, never closed-down during the COVID-19 pandemic.

Public disputes between proponents and opponents of ASRM guidance at times reached unnecessary decibel levels and even resulted in the transient formation of a new interest group of IVF centers with the single purpose of opposing the opinions of the COVID-19 Task Force of the ASRM by creating a competing task force. Though in detail discussed in above-noted Bulletins on the center’s website, we, here, will not further pursue this subject and will address issues of greater importance.

### The role of the “expert”

A subject we, especially during earlier stages of the pandemic, grappled with, was the role of “experts,” in formulating COVID-19 policy. The primary reason was that, for the longest time, none of the “experts” that seemed to determine national and local state COVID-19 policies, ever raised the possibility of unintended consequences from policy interventions implemented at their recommendations during the early days of the pandemic.

This was disturbing because, as physicians, we understood that every medical intervention, before execution, must be carefully weighed for expected benefits and risks. Physicians also understand the additional importance of cost-benefit assessments before interventions. Yet, when the COVID-19 pandemic came upon the nation in March, consequential policy decisions were apparently reached without “experts” (at least in public) ever noting the potential of unintended consequences.

The driving argument at the time was the need to “flatten the curve” of newly infected patients to prevent the collapse of the health care system from excessive demand on hospitals. A perfectly logical motive in early stages of the pandemic, it, however, quickly changed, once initial peaks of the infection had been successfully overcome. “Experts,” now quietly started moving goalposts, without offering clarity as to what the new goalposts were. Advice was at times, indeed, confusing, and sometimes even self-defeating (discussed in the next section in more detail) and, still for some time, continued to fail to address unintended consequences. It was like telling a cancer patient that a therapeutic agent had the potential of curing her cancer without mentioning that the expected mortality was 50%. Unsurprisingly, the public’s trust toward “expert” opinions and governmental decision-making processes started declining.

Our center, indeed, found this situation increasingly ethically challenging. Some “experts” agreed, among those *Stanford* University scientist and professor, John P. A. Ioannidis, MD, who, using data from the cruise ship *Diamond Princess* (it in early February was not allowed to dock in Japan to evacuate COVID-19-stricken passengers and crew) already at very early stages of the pandemic correctly determined infectivity and virulence of the SARS-CoV-2 virus based on infection rates and mortality on the cruise ship. Among 700 passengers, nine deaths were recorded. Ioannidis correctly concluded that the likely fatality rate from the virus was only in the 0.25–0.63% range and, therefore, comparable to the mortality from influenza [24]. Such a low mortality at the time, however, did not fit into the messaging the public was exposed to at by more visible “experts,” and the media. Investigators from the Royal College in London had just predicted two million deaths from the virus in the U.S. alone [25]. Ioannides’ message, and that of a small group of like-minded “experts, was, therefore, largely ignored. To this day unfortunately, public policy on this subject, indeed, appears more influenced by politics than a desire to develop “best” policies for the nation, based on best risk-benefit and cost-benefit outcomes. Exploration of the complex role of “experts” will continue in the next section.

### What it means “to follow science”

A phrase heard over and over during the COVID-19 pandemic was “to follow science.” Politicians of all denominations, media, pundits and even “experts” were constantly laying claim to “following science,” while accusing those with opposing opinions of “denying” or “rejecting” science. We addressed this issue in considerable detail in the June 3, 2020 issue of the *CHR VOICE*, the monthly newsletter of CHR (https://www.centerforhumanreprod.com/fertility/what-does-it-really-mean-to-follow-science/) and are here building on those comments.

To define what it means, “*to follow science*,” one, first must define the meaning of “*science*,” not an easy goal in itself. Merriam-Webster defines science in four ways:

- *“The state of knowing; knowledge as distinguished from ignorance or misunderstanding.”*
- *“A department of systemized knowledge as an object of study.”*
- *“Knowledge or a system of knowledge covering general truth or the operation of general” laws, especially as obtained and tested through scientific methods.*
- *“A system or a method reconciling practical ends with scientific laws.”*

*Google* defines it as:

- *“The intellectual and practical activity encompassing the systematic study of the structure and behavior of the physical and natural world through observation and experiment.”*

*Albert Einstein (1879–1955), in turn, described it as follows:*

- *“It is the fundamental emotion which stands at the cradle of true art and true science*.”

A universally accepted definition of science, therefore, apparently does *not* really exist. One, consequently, is left defining “science” in very personal terms, already pointing out how unlikely everybody means the same thing when claiming “to follow science.” With most scientists fully recognizing how fleeting scientifical knowledge can be, our own definition of science, therefore, emphasizes the limitations of scientific truth to given moments:

- *“The systematic and unbiased pursuit of objective evidence-based truth in the moment.”*

Because science never really offers a “last word,” scientists who believe in absolute certainty must be viewed with a degree of suspicion. Becoming enamored with one’s own truth, defines us as “believers**,”** practicing religiosity, rather than scientists, participating in exploration of the constantly new. “Experts” over time, unfortunately, frequently become believers. Mostly outside of the medical field, roles and trustworthiness of “experts” have, therefore, become an important subject of discussion. Especially the behavioral literature is very clear about the risks of biases in opinions of “experts,” who’s heads are stuck in the sand of highly specialized expertise which prevents them from acknowledging the surrounding landscape.

The philosophy professor Maria Baghramian recently addressed the issue in an assay within the political context of rising anti-elitisms around the world and demagogues asking, “*who needs experts*” [26]? She correctly concluded that we all, of course, do need them since we cannot all be equally knowledgeable about everything. Consequently, we are willing to accept others as sources of authority on matters where they are perceived to know more than we do. In democracies, trust in the advice of “experts,” therefore, becomes essential; yet, is easily withdrawn when experts loose trust of the community because of serious mistakes, dishonesty or obvious biases, as, unfortunately, have permeated “expert”-advice during the COVID-19 pandemic to significant degrees.

Among philosophers, lack of trust in “experts” has, indeed, been especially pronounced when it comes to their roles in policy decisions and is by no means a new phenomenon. Baghramian points out that already the American Pragmatist philosopher, John Dewey, in the early twentieth century raised this issue, to be later in the 1960 followed by the world-famous German-American philosopher and political theorist, Hannah Arendt, who she quotes to have said, *that there are few more frightening things than the steadily increasing prestige of “scientifically minded brain-trusters” in the councils of governments.*

Within this context, Baghhramian further explains that trust in “experts”, of course, requires deference to their opinion; yet, every democratic process is dependent on debate and disagreement, − obviously also a crucially important point in the advancement of medicine and science in general. It, therefore, appears here appropriate to note that “expert-opinion” in medical sciences is universally considered the lowest level of evidence.

Public health and infectious disease experts, of course, must have a seat at the table when policies regarding COVID-19 are developed. They, however, are only meant to offer advisory opinions; they are not meant to determine policy. Under the U.S. constitution, that function is the exclusively privilege of elected officials. They, in turn, have the fiduciary duty of informing themselves as well as is possible by seeking out advice from “experts” with varying opinions. This is where convergence of science and politics amidst the COVID-19 pandemic apparently broke down: Both major political parties ended up lining up their own “experts” in support of their respective own positions. This way, paradoxically, both sides had, indeed, arguments in support of the claim that they were “following science.” Except that most of these alleged science “experts” a long time ago already had become “believers.” Their advice, therefore, can no-longer be viewed as science-based.

“Following science” for a politician does not mean to defer to biased opinions of “believers” but, especially, to seek out also those voices one feels most uncomfortable with. Had this happened, the county’s COVID-19 policies would not have ignored for too long potential unintended adverse effects of implemented policies, and the number of deaths in nursing and other long-care homes would not have represented 41% of the total national COVID-19 mortality, while only representing 8% of national cases [27].

Tragically, COVID-19 could have been managed so much better since no country in the world has more highly qualified “experts” in relevant expertise domains required to reach best policy decisions in unprecedented circumstances, like the COVID-19. We previously noted *Stanford University* professor, John P.A. Ioannidis, MD [24]. That true “expertise” and excellent science can also come from outside medicine was demonstrated by Aaron Ginn, a Silicon Valley technologist, not encumbered by professional preconception and biases [28]. Other experts” from mostly outside of medicine are three economists, *Stanford* University professor Jay Bhattacharya, MD (https://www.wsj.com/articles/the-lockdown-skeptic-they-couldnt-silence-11589566245) from the *University of Southern California,* professors Joel W. Hay, PhD, a professor of Pharmaceutical and Health Economics (https://healthpolicy.usc.edu/author/joel-w-hay-ph-d/) and, finally, professor Neeray Sood, PhD at the same school (https://priceschool.usc.edu/people/neeraj-sood/).

### What are essential workers/services?

We previously addressed our center’s position that fertility services, if time-dependent, do represent essential services. Those who provide these services, therefore, are essential workers. As also noted earlier, our interpretation of infertility services being “essential” was reaffirmed in N.Y. state by official proclamation of the governor [23].

This is, however, not how much of the infertility field viewed itself during the pandemic. Reflected in guidances from ASRM (https://www.asrm.org/news-and-publications/news-and-research/press-releases-and-bulletins/asrm-issues-new-guidance-on-fertility-care-during-covid-19-pandemiccalls-for-suspension-of-most-treatments/), and even more so from ESHRE (https://www.eshre.eu/Press-Room/ESHRE-News#COVID19_April2), we were surprised where the field’s major professional societies perceived infertility services to rank in importance. Concerned about overwhelming the health care system if all possible resources were not concentrated on the pandemic, both organizations, obviously, did *not* consider most fertility services essential medical services. The self-esteem of the profession in its medical and biologic importance, therefore, appears low, and is especially surprising, considering that reproduction leads the hierarchy of evolutionary survival functions of all species.

Claiming infertility as a disease, warranting third-party insurance coverage as other diseases did [29], this low self-appreciation also seems surprising on a more practical level. It, moreover, also explains why others see the importance of the fertility field in similar ways. The contrast in third-party funding of reproductive sciences and reproductive clinical care by governments as well as private foundations is, likely, the most striking example. It is best documented by comparing benefits of infertility treatments for society, to benefits of end-of-life care: While every successful infertility treatment results in a live birth and lifelong benefits to society, end-of-life care consumes most health care dollars with, of course, much shorter-term benefits for society. Yet, there is not even a reasonable comparison to be made when it comes to financial support between these two areas of medicine because support of reproductive medicine, of course, dwarfs that of end-of-life care.

Unsurprisingly, the low standing of infertility services within the medical hierarchy is also shared by the general public [30], though, of course, likely not by individuals affected by infertility. They, however, represent only a small minority. Considering how we view ourselves as a medical specialty, we cannot be surprised that most of the public sees us in similar terms. This should be cause for concern in the leadership of the field’s professional organizations and lead to a self-evaluation process that better defines the field.

## To be pregnant during COVID-19

After the 2015 Zika virus epidemic in Brazil was associated with severe adverse effects on pregnancy and offspring, including miscarriages and preterm birth as well as the congenital Zika syndrome (microcephaly and other congenital malformations) [31], a viral pandemic, understandably, created concerns about potential effects the SARS-CoV-2 virus might have on pregnancy and offspring. Those concerns were further strengthened by other respiratory viruses, especially influenza, which appears, as is known, to increase hospital admissions in pregnant women, though does not increase maternal mortality [32].

Despite an abundance of recent publications, effects of the SARS-CoV-2 virus on pregnancy and offspring are, still, not fully elucidated. Some studies, including a systematic review of U.S. data by CDC [33] and a small case series from China [34] suggest similar clinical severity in pregnant and non-pregnant infected women. A more recent CDC report, however, went into more detail [35]: Among 91,412 women with laboratory-confirmed infections, 8207 (9.0%) were pregnant. Prevalence of cough and shortness of breath were similar between pregnant and non-pregnant women; pregnant women, however, reported less headache, muscle aches, fever, chills and diarrhea. Pregnant COVID-19 patients were, in contrast, more frequently hospitalized (31.5%) than non-pregnant women (5.8%). Because this could be attributed to preferential treatments given to pregnant women and/or the fact that they demonstrated more chronic lung disease, diabetes mellitus and cardiovascular diseases, investigators adjusted data for age, underlying medical conditions and race/ethnicity and, still, found that pregnant women with COVID-19 were more frequently admitted into intensive care (aRR = 1.5, 95%CI = 1.2–1.8) and received more frequently mechanical ventilation (arr = 1.7,95%CI = 1.2–2.4). Death rates between ages 15–44 were, however, identical at only 0.2%, − a reassuring finding.

Similarly, to influenza, it, thus, appears that pregnancy may aggravate certain manifestations of COVID-19 but does so without increasing mortality risks. In a study involving five major medical centers in NYC, among 241 pregnant women with SARS-CoV-2 infections admitted to the hospital for labor and delivery, a majority (61.4%) were asymptomatic. Throughout their hospitalization, 26.5% ultimately met criteria for mild, 26.1% for severe and only 5% for critical disease [36]. Similarly, to non-pregnant individuals, pregnant women with COVID-19 also revealed racial/ethnic inequities: A Boston study demonstrated that 39/54 (72%) of Hispanic but only 22/82 non-Hispanic women (27%; *P* < 0.001) had positive viral tests. Severity of disease did not appear to differ [37]. In Illinois, Black and Hispanic women represented among tested individuals over 70% of positive COVID-19-cases [38].

Like most infectious diseases, COVID-19 can adversely affect pregnancy outcomes: Preterm birth rates have been reported 20.1%, and cesarean section rates at 84.7% [39]. Though several case reports have suggested maternal-fetal transmission of the virus during pregnancy, such cases are extremely rare [40, 41]. If transmission occurs, it, likely, is linked to severe placental damage from underlying maternal disease conditions. Under normal circumstances the SARS-CoV-2 virus, therefore, does not appear to cross the placental barrier into the fetus. That, indeed, may the best news regarding the virus we here can report.

## The future post COVID-19

The COVID-19 pandemic has already affected the whole world. The infertility field, of course, cannot avoid consequences. As this manuscript is written, the U.S., still, is in midst of a second wave of infections, mostly encompassing south western states. Modelers just warned that Baltimore, Boston and Chicago may become the next COVID-19 hot-spots [42]. More recent evidence supports that phenotype C states, mostly in the Midwest may become the last affected region. We, currently, expect most U.S. states to reach an adequate level of combined (innate and adaptive) immunity sometimes between the end of November and December. This does not mean that after that point there will be no more COVID-19 cases in the U.S.; but they, in our opinion, will be relatively uncommon, as long as minimal distancing and face-masking is maintained, likely, at a level similar to what NYC has been experiencing in recent months (~1% test-positivity).

In looking at the future impact of the currently still ongoing pandemic, one, therefore, must bifurcate into short- and long-term consequences as well as into general societal consequences and specific consequences for the fertility field: On a societal level, consequences will, likely, be substantial in almost all aspects of society, with the ultimate length of the pandemic playing a major role in determining medium- and long-term effects on health, economy and politics. For example, the longer small businesses will have to remain shut-down, the less likely will they ever reopen. General economic consequences are, however, not the subject of this manuscript. When it comes to seeking out, receiving and providing fertility services, which is subject of this manuscript, we are already witnessing some of the short-term consequences and can only hypothesize about the longer term.

As most fertility centers have by now reopened and are again providing comprehensive infertility services, each center does so distinctively differently from pre-pandemic times. As circumstances have changed, professional societies have continued to publish ever-changing guidances, with ASRM, ESHRE and the International Federation of Fertility Societies (IFFS) most recently publishing an interesting joint statement that reemphasizes a “*joint affirmation of the importance of continued reproductive care during the COVID-19 pandemic*” [43].

The statement reflects a subtle evolution of our profession’s self-assessment, as earlier described. Reaffirming reproduction as a basic human right, the statement also defines infertility as *“a serious disease” .... “that harms physical and mental well-being.”* The statement also points out that, *“infertility is time-sensitive, and prognosis worsens with age.”* Maybe even most interestingly, the statement claims that “*ASRM and ESHRE, independently, recommended discontinuation of reproductive care* (during early stages of the pandemic) *except for the most urgent cases*.” This was, indeed, our understanding of ASRM’s initial guidance (though we were unable to interpret ESHRE’s initial statement in this way).

Remarkably, one paragraph in the joined statement carries the heading, “*reproductive medicine is essential*.” This, of course, is exactly the point we and selected other colleagues have made before; but, considering the almost worldwide closing-down of fertility services in early stages of the pandemic, one has to wonder why the essential nature of at least urgent fertility services was not acknowledged more clearly before. Finally, the statement of the three societies also notes that, “*reproductive care is essential for the well-being of society and for sustaining birth rates at a time when many nations are experiencing declines*.”

### How COVID-19 will affect the future of the infertility field

Which brings us to a very recent publication from the Guttmacher Institute which just published a report on the early impacts of the COVID-19 pandemic on reproductive health experiences [44]. Some of the findings were eye-opening and suggest that changes may be broader and more significant than most of us currently appreciate. In an assessment of different aspects of reproductive health, the report reemphasized race-dependent inequalities in COVID-19 infection rates, differences in mortality and job losses and that large increases in unemployment often result in loss of health insurance and, therefore, loss in access to health care. Based on an Internet survey, the investigators furthermore reaffirmed what has been repeatedly demonstrated: Fertility preferences must be viewed within a broad socioeconomic context.

Over 40% of women reported to have changed their plans about when to have children and how many children they were desirous of. Overall, 34% of women indicated that they would delay childbirth and/or would have fewer children. With considerable relevance to the infertility field, somewhat unexpectedly, these changes in preference were more common among still childless women (45%) than women who already had children (38% (*P* < 0.05), suggesting a significant degree of potential shrinkage in the market for fertility services, at least in near- to medium-future.

We in the past investigated IVF cycle numbers preceding, during and following the severe economic recession in 2008, and noted a very significant decline that carried over for considerable time beyond the end of the recession [45]. Considering the severity of the current economic downturn, the fertility field, therefore, must be ready for an extended and rather severe draught in IVF cycle activity. Also unsurprisingly, Black (44%) and Hispanic women (48%) voiced these sentiments more frequently than Caucasian women (28%) and lower income resulted in higher percentages (37%) than higher incomes (32%). All cited differences were statistically significant at least at P < 0.05 levels.

How significant the financial impact will be on the infertility industry is, still, difficult to predict. We, however, expect it to cause major realignments. Those will, likely, be further exacerbated by rapid expansion in the number of IVF centers worldwide in recent years, mostly driven by outside equity investments, until recently considered by the financial industry to offer exceptional investment opportunities [46]. Since such investments usually have relatively short cash-out horizons, we would expect serious reconsiderations regarding such investments in coming months and years. We would also foresee early-cash outs, mergers, fire sales and even bankruptcies. The country’s oldest publicly held company in the infertility field, IntegraMed America, indeed, recently already filed for Chapter 7 liquidation [47].

Significant changes in the infertility field can, however, also be expected in how fertility services will be provided. A group of mostly European colleagues recently published in this journal a proposal for individualized clinical management of assisted reproductive technology services in a COVID-19 environment [48]. The routine face-to-face consultation, which represented a large majority of patient-physician encounters in all of medicine, has been largely replaced by electronic platforms. It is difficult to imagine that this change in practice pattern will not have long-term effects. Because of COVID-19, physicians also no longer are restricted from electronic patient contacts across state-lines. The need for individual state licensures may, therefore, also have outlived its usefulness. In infertility practice, where “patient tourism” is common, this may have special relevance. At our center, the switch from personal to electronic contacts involved not only physicians but also nurses and other staff. Like in other industries, this will have significant economic consequences on employment, on space needs and, of course, on overall management structures. Since, at least in the short-term, we anticipate a degree of financial hardship for most fertility centers, most will be forced to provide services to patients in more economic ways.

## Conclusions

To be a fertility center during COVID-19 in NYC has been for several reasons a unique, unprecedented and highly consequential experience. For our center, this experience was based on an unprecedented pandemic, consequentially severe social and economic disruptions, political unrest and, finally, demonstrations and even riots in the streets coming dangerously close to our center. What differentiated our center, however mostly, was that, in contrast to most other fertility centers in NYC, we never closed. Our center, therefore, did not have the benefit of following advice on how to manage an infertility center during a pandemic but was forced to invent a completely new practice format based on intuition and constant analysis of published literature as, slowly, at least limited information about the SARS-CoV-2 virus started to emerge.

Maybe somewhat surprisingly, this ended up becoming a highly rewarding experience for physicians, most staff and patients. At the same time, we also must acknowledge how lucky we have been so-far in avoiding the virus-contaminating our center. Finally, it was remarkable to learn how quickly one can familiarize oneself with a completely new field in medicine when there, simply, is no other choice.

## Addendum in proof

A study published after completion of this manuscript confirmed the in this manuscript presented hypothesis that the SARS-CoV-2 variant carrying the Spike protein amino acid change G614 has taken over from the D614 variant on national, regional and municipal levels all over the world due to a likely fitness advantage, even where the original D614 form had been well established before [49]. The study also provided further evidence for the increased infectivity of G614 but, interestingly, did not demonstrate increased severity of disease. An accompanying editorial attempted “to make sense” of these findings, concluding that the evidence for G614 being more infective is compelling, though not conclusive, while evidence for increased virulence of G614 apparently does not exist [50].

These findings then raise the question why the mortality in A states during early stages of the pandemic was so much higher than in B and C states (Table 2)? Moreover, during the currently still ongoing later wave, despite high infection rates, mortality rates have remained low in affected states, more akin to B- than A states during the early wave. The only explanation for these findings we can come up with is that G614, indeed, is not only more infectious but, likely, also more virulent. Its virulence is reflected in originally higher mortality rates in A than B and C states. Mortality, however, depends on the existing immunity background in a community. A states during the early stages of the pandemic had very little immunity and, therefore, likely, faced the full force of the G614 variety of the virus. South western states initially faced the less-infective and less-virulent D614 strain yet were able to build up a degree of anti-SARS-CoV-2 immunity. By the time those regions were taken over by the G614 strain a certain level of community immunity already was protective and reduced the virulence of the virus. This hypothesis, of course, requires further experimental confirmation. The recent publication by Korber et al. [49], however, clearly validates our in this manuscript first-time presented epidemic state phenotypes A, B and C.
